# Micro- and nano-structural details of a spider's filter for substrate vibrations: relevance for low-frequency signal transmission

**DOI:** 10.1098/rsif.2014.1111

**Published:** 2015-03-06

**Authors:** Maxim Erko, Osnat Younes-Metzler, Alexander Rack, Paul Zaslansky, Seth L. Young, Garrett Milliron, Marius Chyasnavichyus, Friedrich G. Barth, Peter Fratzl, Vladimir Tsukruk, Igor Zlotnikov, Yael Politi

**Affiliations:** 1 Department of Biomaterials, Max Planck Institute of Colloids and Interfaces, Research Campus Golm, 14424 Potsdam, Germany; 2 European Synchrotron Radiation Facility, 38043 Grenoble, France; 3 Charite, Berlin Brandenburg Center for Regenerative Therapies, Julius Wolff Institute, 13353 Berlin, Germany; 4 School of Materials Science and Engineering, Georgia Institute of Technology, Atlanta, Georgia 30332, USA; 5 Department of Neurobiology, Faculty of Life Sciences, University of Vienna, 1090 Vienna, Austria

**Keywords:** chitin, mechanosensing, spiders

## Abstract

The metatarsal lyriform organ of the Central American wandering spider *Cupiennius salei* is its most sensitive vibration detector. It is able to sense a wide range of vibration stimuli over four orders of magnitude in frequency between at least as low as 0.1 Hz and several kilohertz. Transmission of the vibrations to the slit organ is controlled by a cuticular pad in front of it. While the mechanism of high-frequency stimulus transfer (above *ca* 40 Hz) is well understood and related to the viscoelastic properties of the pad's epicuticle, it is not yet clear how low-frequency stimuli (less than 40 Hz) are transmitted. Here, we study how the pad material affects the pad's mechanical properties and thus its role in the transfer of the stimulus, using a variety of experimental techniques, such as X-ray micro-computed tomography for three-dimensional imaging, X-ray scattering for structural analysis, and atomic force microscopy and scanning electron microscopy for surface imaging. The mechanical properties were investigated using scanning acoustic microscopy and nanoindentation. We show that large tarsal deflections cause large deformation in the distal highly hydrated part of the pad. Beyond this region, a sclerotized region serves as a supporting frame which resists the deformation and is displaced to push against the slits, with displacement values considerably scaled down to only a few micrometres. Unravelling the structural arrangement in such specialized structures may provide conceptual ideas for the design of new materials capable of controlling a technical sensor's specificity and selectivity, which is so typical of biological sensors.

## 1. Introduction

Mechano-sensing is ubiquitous in nature. Examples are hearing, tactile and vibration sensing, the sensing of air and water flow, strain and stretch. Mechano-sensing in arachnids, in general and in spiders in particular, is well recognized for its high sensitivity and specificity [1–3]. The spiders' strain detectors, the so-called slit sensilla, are elongated openings within the exoskeleton, innervated by sensory cells and often located in the vicinity of joints in the legs or elsewhere [4–7]. They are widespread among spiders and share some similarity to the campaniform strain sensors of insects. The metatarsal lyriform organ (HS10) of the Central American wandering spider *Cupiennius salei* forms a close and roughly parallel array of 21 slits. It is the spider's main vibration detector [8–10]. It senses vibrations originating from predators, prey or courting partners with extraordinary sensitivity [11,12]. These vibration stimuli are usually of comparatively high frequency (40 to several hundred hertz). In addition, the metatarsal lyriform organ responds to low-frequency vibrations (0.1–40 Hz) with nervous impulses, although with much lower sensitivity [8]. Such low-frequency stimuli occur during locomotion and other activities. Taken together, the range of frequencies detected by the slits spans as much as four orders of magnitude as also shown for another lyriform organ (HS8) [13].

**Figure 1.**
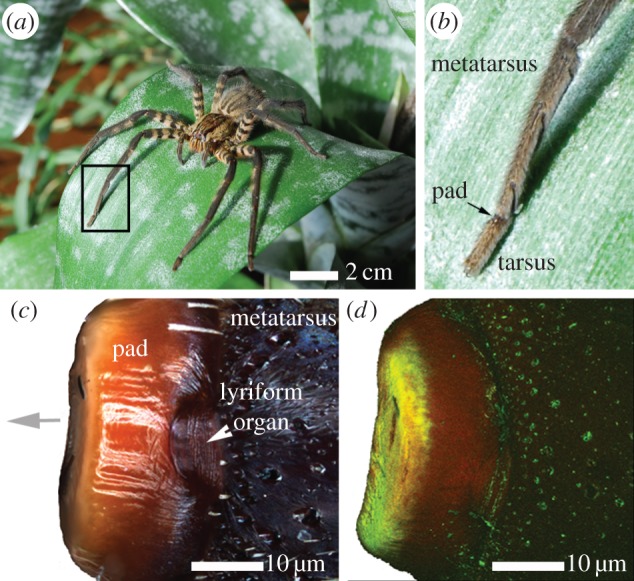
(*a*) Adult female spider *Cupiennius salei*. (*b*) Magnification of the two last (distal) leg segments; the metatarsus and the tarsus. The cuticular pad is situated at joint between the two segments (arrow). (*c*) Optical light microscope image of the cuticular pad and the vibration receptor of the spider. Top view on the dorsal side of the pad. The distal direction is marked by a grey arrow. White arrowhead indicates vibration-sensitive metatarsal lyriform organ. (*d*) CLSM view of the pad in (*c*). The image is constructed by a superposition of the auto-fluorescence signals (as maximum intensity projection) of excitation/detected emission wavelengths: 488/499–555 nm (green channel); 561/578–678 nm (red channel).

HS10 lyriform organ is situated at the distal end of the second last segment of each leg, i.e. their metatarsus (figure 1). Substrate vibrations deflect the most distal leg segment, the tarsus, which in turn transmits the signal to the vibration-sensitive slit sensilla by pressing against the metatarsus. A cuticular pad (figure 1*b*–*d*) is located just in front of the slit lyriform organ, at the contact area with the tarsus. The pad plays a major role in the mechanical filtering of vibrational stimuli by forming an effective high-pass filter [14]. This latter function was recently mapped to the external most layer of the pad, the epicuticle [15]. This layer is unusually thick and behaves visco-elastically with the glass transition temperature of around 19°C [15].

In its natural habitat and during its time of activity, the spider sits on a leaf in its characteristic ‘hunting position’, with the proximal end of the tarsus just in contact with the pad. From this point of contact onwards, further deflection of the tarsus will elicit action potentials in the sensory cells of the metatarsal lyriform organ—if its amplitude is above the threshold (depending on the frequency) and overcomes the filtering restrictions of the pad. Electrophysiological studies have shown that for high frequencies only minute deflections of the tarsus beyond the ‘first contact’ with the pad are needed in order to elicit action potentials. In particular, for frequencies of several hundred hertz vertical displacements of the substrate down to the order of nanometres and corresponding to deflection angles of around 0.01° from the ‘first contact’ angle elicit physiological responses in the receptor cell [8,16]. Below stimulus frequencies of about 40 Hz, however, threshold deflections of the tarsus by tens of micrometres or up to 10° from the ‘first contact’ position are needed in order to elicit a nervous response.

The remarkably high sensitivity of the slits at high frequencies is owed first to the mechanical properties of the epicuticular layer of the pad, which exhibits a glassy state at these frequencies, thus efficiently transmitting the signal [15]. However, other mechanical factors affecting the mechanical sensitivity of the slits at a subsequent stage of stimulus transmission are slit length, aspect ratio and specific location within the array of slits as previously documented in detail [7,17,18]. The question remains: how are low-frequency stimuli transmitted to the slits considering that the epicuticle then exhibits a rubbery state, strongly damping the incoming vibrations? In addition, since large deflection amplitudes are involved, what protects the delicate slit system from mechanical damage?

Here we address these questions by studying the structure and composition of the pad and relating them to the material's mechanical properties. We also monitor the three-dimensional deformation behaviour of the pad upon the static application of mechanical load similar to the natural load application at low frequencies. Thus, the tarsus was deflected against the pad during X-ray micro-computed tomography (µCT) measurements in wet state. We show that the three-dimensional morphology and specific sub-structure of the pad allows large tarsal deflections to be converted into small displacements at the pad-slit contact region, thus allowing transmission of low-frequency stimuli while providing mechanical robustness and damage protection at high loads.

## 2. Material and methods

### 2.1. Sample preparation

Adult females of the Central American wandering spider *C. salei* [1] were received from the breeding stock of the Department of Neurobiology of the University of Vienna. In all experiments, except in those applying µCT, we used spider legs taken from shock frozen (at −196°C) specimens initially anaesthetized and kept at around −18°C until use. For µCT experiments, fresh autotomized spider legs were prepared. For a better view, the hairs covering the metatarsus were removed by gently wiping the surface with a cotton tissue. The most distal two leg segments of freshly autotomized spider legs were separated from the rest of the leg using a scalpel. In order to avoid drying, the fresh cut was instantly sealed with a droplet of candle wax.

#### 2.1.1. Sectioning

For X-ray scattering experiments and for confocal laser scanning microscopy (CLSM) the pad was dissected from the tarsus and sectioned into 30 µm thick slices using a vibratome (Leica VT1000S; Leica Microsystems GmbH, Wetzlar, Germany) at 80 Hz steel blade frequency and 0.025 mm s^−1^ blade velocity. For sectioning, the metatarsus was glued to a plastic substrate using a tiny superglue droplet (Roti coll 1, Carl Roth, Karlsruhe, Germany). Contamination of the pad region with the superglue was avoided by fixing the metatarsus sufficiently away from its distal side where the pad and the slits are situated. The slices were cut in high-purity water at 20°C and kept wet until the time of the experiment. The entire sample preparation process took only a few minutes and was performed shortly before each measurement.

#### 2.1.2. Embedding and polishing

For scanning acoustic microscopy (SAM) measurements and for nanoindentation the metatarsus containing the pad was immersed in methylmetacrylate (MMA) for 8 h followed by polymerization in an oven at 60°C. The embedded samples were gently polished (in their sagittal plane) until the cuticular pad region of interest was exposed to the surface. Because incubation in MMA and heating to 60°C cause drying of the samples, after sectioning and polishing the samples were rehydrated (considerable swelling was observed) in order to measure the properties of the sample as close as possible to their natural state. We expect limited influence of the embedding material (reduced elastic modulus of 4 GPa) on the measured mechanical properties of the cuticle since we do not observe significant infiltration of MMA into the sample as judged from the fact that the sample can be easily detached from the embedding material and that the swelling behaviour is not affected.

### 2.2. Optical microscope imaging

Optical images were taken using a light microscope equipped with a digital camera (Dage-MTI XLM high-resolution, cooled) at 10× magnification under dark field illumination. The clarity of each optical image was digitally enhanced by focus stacking using Helicon Focus v. 5.3.14 software. Hereby, more than 100 images recorded at different focal planes were superimposed giving a single high-resolution image.

Polarized light images (1388 × 1038 pixel; 0.37 µm pixel^−1^) were recorded on an upright microscope (Axio Imager A2, Zeiss, Jena, Germany) equipped with a 20× objective (LD A-Plan 20×/0.35 Ph2) (Zeiss) using a polarizer and an analyser with a relative orientation of 90° to each other. The images were analysed using the software package Zen 2012 (Zeiss).

### 2.3. *In situ* synchrotron-based micro-computed tomography

The experiments were performed at the European Synchrotron Radiation Facility (ESRF) in Grenoble, France, using the imaging set-up at beamline ID19. In order to find a compromise between high imaging sensitivity and low radiation damage, the samples were measured at 26 keV photon energy [19]. A so-called single-harmonic undulator (u13, gap 11.5 mm) with a narrow bandwidth was used as radiation source with a diamond filter and a Be window as the only mandatory optical elements in the beam path, leading to a homogeneous wavefront at the position of the experiment and therefore excellently suited for X-ray phase contrast imaging in parallel-beam geometry. Approximately 35 mm downstream of the specimen a high-resolution indirect imaging detector was placed. It was equipped with a 8.8 µm thin single-crystal scintillator (Tb-doped Lu_2_SiO_5_), a 10× objective (0.3 numerical aperture), a 2× eye-piece and the ESRF custom made CCD camera FReLoN (type: A7899), operating with a nominal effective pixel size of 0.7 µm [20]. The exposure time was set to 0.2 s for each of 1000 projection images while rotating the sample through 180°. Pad morphology data were reconstructed using the ESRF software package PyHST_2 [21], which includes a phase-retrieval using Paganin's approach [22]. For data visualization and segmentation, ZIBAmira software (Zuse Institute, Berlin, Germany; FEI Visualization Science Group, Burlington MA, USA) was used.

#### 2.3.1. Tomo-press

The micro-tomography fatigue press (Tomo-press) developed at ESRF was originally designed to scan samples under axial load such as bone in order to understand crack propagation [23]. It was employed here to static load the tarsus and to stimulate a natural low-frequency and high-amplitude substrate displacement. The fresh autotomized spider samples now contained only the metatarsus and the tarsus. The metatarsus was fixed horizontally on one side of the Tomo-press while the free-hanging tarsus was pointing towards the opposite side, where a rectangular aluminium wedge was mounted. Using a remote control, the wedge was brought closer towards the tarsus thus pushing it against the pad as in natural stimulation. The desired deflection angle between the tarsus and the metatarsus (between 0° and 45°) was adjusted by horizontally changing the distance between the two fixed parts. The corresponding vertical displacement of the tarsus distal side (substrate displacement) was calculated by measuring the angle between the two leg segments from digital images of the camera connected via a C-mount to a microscope with long working distance. A series of rotation angles was used to avoid perspective errors. The maximum angle value of 45° was defined by the geometry of the aluminium wedge. The angular deflection values were chosen according to previously reported values well within the physiological working range of the slits [16,17]. The tarsus was thus deflected against the metatarsus in a stepwise manner, and µCT datasets were obtained at each stationary position after 1 h relaxation time for each step. For tomography measurements, the sample was rotated around the horizontal axis while keeping the distance between the leg sample and the wedge constant. The Tomo-press chamber was sealed with Kapton foil and was kept humid by a continuous stream of fully hydrated air during the entire course of the measurements. Two tarsus–metatarsus joints were measured, at six and at three angular positions, respectively. The temperature inside the hutch was maintained at 20°C, which is above the glass transition temperature *T*_g_ of the epicuticle [15].

### 2.4. Electron microscopy

Scanning electron microscopy (SEM) images were taken in high vacuum (7.5 × 10^−8^ Torr) using a field emission scanning electron microscope JEOL JSM-7500F in low-magnification mode. The pad samples were mounted on an SEM sample-holder with conductive carbon tape. The measurements were performed at a working distance of 11.6 mm and at 5.0 kV.

### 2.5. Atomic force microscopy

Atomic force microscopy (AFM) imaging was conducted using a Bruker Icon (Bruker, Santa Barbara, USA) with a Nanoscope V controller. Topographical images were taken using peak force tapping mode (PFT) in Bruker's Quantitative Nanomechanics module. In PFT-mode, a cantilever is oscillated sinusoidally at 2 kHz, and briefly contacts the sample surface at the downstroke of each cycle. A user-established set point force is established such that the sample surface is minimally deformed (on the order of a few nanometres) and used as feedback control. Scanned images were taken at 512 × 512 pixel resolution with scanning rates less than 1 Hz.

### 2.6. Confocal laser scanning microscopy

Free-standing pad sections (30 µm thick) were imaged on a Leica TCS SP5 (Leica Microsystems GmbH, Wetzlar, Germany) equipped with an inverted microscope (Leica DM IRBE) and two visible light lasers (wavelengths 488 nm, argon, 100 mW; 561 nm, diode pumped solid-state DPSS, 10 mW). For each sample, the most appropriate lens was chosen depending on sample size. The excitation wavelengths and the wavelengths of the emitted fluorescence were chosen according to previous studies of arthropod cuticle [24]. The power of each laser was reduced to 2 mW to avoid damage of the sample. The signal detection gain was optimized for each sample individually. The lenses and settings used for the visualization of each sample are given within the respective figure caption. Image data processing was performed using ImageJ software [25]. The auto-fluorescence images represent superimposed emission signals resulting from 488 nm and 561 nm excitation wavelengths assigned to the green and the red software channel, respectively.

### 2.7. Synchrotron-based X-ray scattering

X-ray scattering experiments were performed at the µ-spot beamline at the BESSY II synchrotron radiation facility in Berlin, Germany. The wavelength of 0.826 Å (energy 15 keV) was selected using a multilayer monochromator. The beam was focused by a toroidal mirror and the scattering patterns were collected on the area detector (MarCCD 225, MarUSA, Evanston) with 3072 × 3072 pixels placed behind the sample. The sample-detector distance was 0.408 m and the beam diameter at the sample position was 10 µm. This gives a covered range of scattering vectors of 0.4 nm^−1^ < *q* < 25 nm^−1^, defined by *q* = 4*π* sin (*θ*)/*λ*, where 2*θ* is the scattering angle and *λ* the wavelength of the incident beam. All measurements were calibrated using a quartz powder placed at the sample position. For the analysis of the two-dimensional scattering data, the software Fit2D [26] was used.

### 2.8. Scanning acoustic microscopy

SAM measurements were performed using KSISAM2000 by Krämer Scientific Instruments (Herborn, Germany), and data were collected using software MATSAM custom made by the Q-BAM Laboratory (University of Halle-Wittenberg, Germany). Temperature in the room was maintained at 21°C. Two spider leg samples had been previously embedded in MMA. After polishing (removing one half of the metatarsus) the sample surface represented the sagittal plane of the pad. The SAM analysis of this surface was performed under deionized water using 400 Hz and 820 Hz, respectively.

### 2.9. Nanoindentation

Nanoindentation measurements were performed using Ubi1 Nano Indenter (Hysitron, Minneapolis, MN, USA). The same samples were used as for SAM. After choosing regions for measurements using a built-in light microscope, the samples were immersed in water and allowed to equilibrate for 30 min. In this time, the samples swelled considerably. The measurements were performed under water with the Berkovich tip immersed in deionized water. The following load function was applied: loading/unloading rates: 100 µN s^−1^, holding time 60 s at a peak load of 500 µN. Each measurement included 64 indents at the respective pad region. The values for the reduced elastic modulus *E*_r_ and for hardness *H* were obtained from the load–displacement curves according to the Oliver and Pharr method [27]. During measurements, the temperature was kept at 24°C, well above the glass transition temperature of the epicuticle [15].

## 3. Results and discussion

### 3.1. Pad morphology and deformation during load

**Figure 2.**
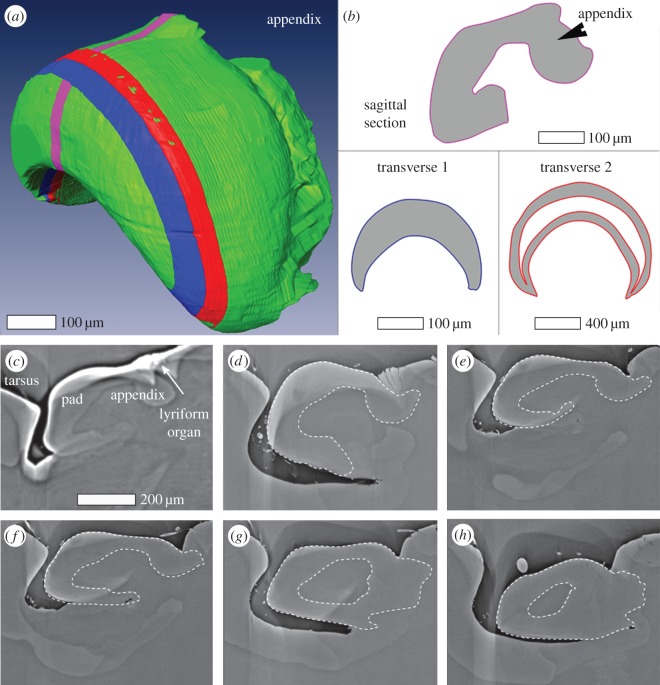
(*a*) Surface rendering of the reconstructed µCT data of the pad. Virtual sections representing sample sections used in this study are indicated by three differently coloured slices: pink, blue and red (*b*) Schematic of the shape for the slices shown in (*a*). (*c*–*h*) µCT virtual slices along the long axis of the leg (sagittal plane) laterally extending from the pad centre (*c*) to the pad lateral edge (*h*). The dashed lines show the outline of the pad traced along the organ. The line was determined using a number of successive images.

The three-dimensional shape of the pad is rather complex (figure 2): it is crescent-like and its lumen is filled with cellular materials showing slightly lighter contrast in the µCT data. Just below the lyriform organ a small cuticular ‘ridge’ extends along a plane perpendicular to the leg long axis, which is roughly parallel to that of the slits. We refer to this ridge as the ‘appendix’. Figure 2*a* shows the pad surface according to µCT data (green) with three sub-regions marked in different colours. These regions specify the location and orientation of three types of sections used in our compositional and structural analyses. The outlines of each section are also depicted schematically in figure 2*b*. In figure 2*c*–*h*, six sagittal virtual sections of the reconstructed three-dimensional pad structure (extending laterally from the centre of the pad) illustrate its structure.

**Figure 3.**
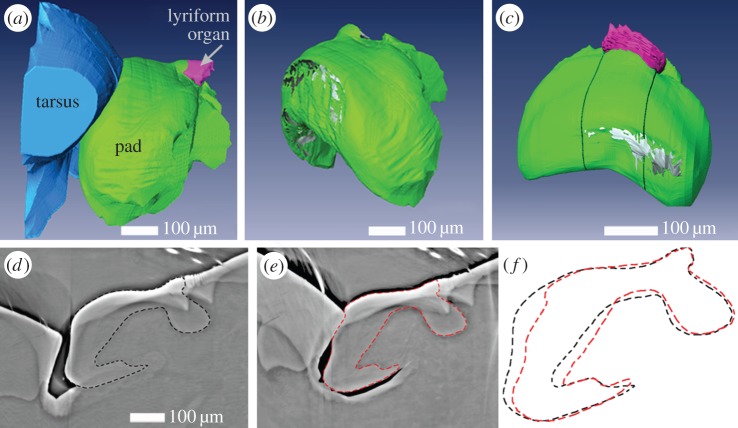
(*a*) Surface rendering of reconstructed µCT data showing three selected components of the metatarsal vibration receptor including the tarsus (blue), the pad (green), and the slit-sensilla lyriform organ (pink) measured during contact. The deflection angle between the tarsus and metatarsus was 9°. (*b*) Three-dimensional shape of the cuticular pad extracted from (*a*). Grey regions at the distal side of the pad indicate the contact area with the tarsus. (*c*) Three-dimensional shape of the cuticular pad under load with a slight lateral component. The tarsus–metatarsus angle was 8°. Grey regions at the distal side of the pad indicate the contact area with the tarsus. (*d*–*f*) µCT virtual slices of the sample in a–b sectioned in the sagittal plane in the centre of the pad in relaxed (*d*) state (less than 0°), and deflected by 9° (*e*). The dashed lines indicate the outline of the cuticular material of the pad. The white arrows indicate one slit of the metatarsal lyriform organ. Darker region below the ventral side of the pad is caused due to reduction in vapour pressure; the pad itself however is still moist. (*f*) An overlay of the pad shape from *d* and *e*.

In order to better visualize and quantify the deformation of the pad under loads relevant to the transmission of low-frequency stimuli to the slits, we performed X-ray micro-tomography measurements (under hydrated conditions) of the pad in a compressed state, stimulated by deflecting the tarsus against the pad. The angle formed by the long axes of tarsus and metatarsus at which the tarsus first touches the pad is defined as the ‘mechanical threshold angle’ [8,16] and measures 28° (±1°). From now on, we refer to this angle as 0° when deflecting the tarsus against the metatarsus. We measured six different angular positions between −4° and 9° (±1°). The corresponding µCT virtual slices along the long axis of the leg are given in electronic supplementary material, figure S2. For angles above 0°, the tarsus pushes into the pad, significantly deforming its distal part. On the tarsus side, on the other hand, we did not observe any deformation. We quantified the pad deformation by measuring the distance of the distal surface of the pad at each degree from its resting position at 0° (table 1). Thus, for example, at 9° ± 1°, the pad surface is pushed in 29.4 ± 0.7 µm. Figure 3*d*–*e* shows that other parts of the pad hardly change their shape. However, small displacements of a few micrometres (3.5 ± 0.7 µm at 9° ± 1°) at the contact region with the slits can be seen. In addition, the slits themselves are also compressed by up to 50–60% of their initial width (width of the most distal slit at 9° ± 1° in figure 3*d*–*e*), which is in agreement with a previous white light interferometry study [17]. Importantly, the slits are compressed evenly throughout their length and depth. Another interesting observation from figure 3*b* is that the contact area of the tarsus and the pad is limited to a rather small region. When the pad is loaded by a lateral deflection, the lateral regions of the pad are the ones that are maximally deformed (figure 3*c* and electronic supplementary material, figure S2). Such deflections do occur during natural stimulations and are also known to elicit action potentials [1].

**Table 1.** Parameters for the static loading of the pad following µCT measurements (sample without lateral deflection component). Deflection angle values were extracted from the microscope image analysis. The substrate vertical displacement was calculated from the distance adjusted between the Tomo-press mounting tools (fixing the metatarsus on the one side and the aluminium wedge on the other side). The pad displacement and deformation values were extracted from the µCT data analysis.

deflection angle between tarsus and metatarsus (°) error (±1°)	tarsus distal side (substrate) vertical displacement (µm) error (±5 µm)	pad deformation at contact region with the tarsus (µm) error (±0.7 µm)	pad displacement at contact region with the slits (µm) error (±0.7 µm)
0	0	0	0
2	66	8.4	0
4	111	14.7	0.7
7	150	23.8	2.1
9	181	29.4.	3.5

### 3.2. Pad sclerotization pattern

Figure 1*c* shows the dorsal view of an intact pad. The different colours of the distal and dorsal sides of the pad suggest different degrees of sclerotization, the darker colour often being associated with higher sclerotization [28]. While the surface is bright creamy-coloured distally (grey arrow in figure 1*c*), it is darker dorsally. Auto-fluorescence measurements performed with a CLSM and two different excitation wavelengths support the assumption regarding the different degrees of sclerotization. In figure 1*d*, the emission signal images collected at 499–555 nm (green channel) and 578–678 nm (red channel) for excitation at 488 and 561 nm, respectively, are overlaid to form one composite image. Whereas the distal surface of the pad shows a predominantly green channel auto-fluorescence signal, usually attributed to cuticles with low sclerotization levels, the dorsal surface shows a strong auto-fluorescence signal in the red channel, indicative of stronger cuticle sclerotization [29].

**Figure 4.**
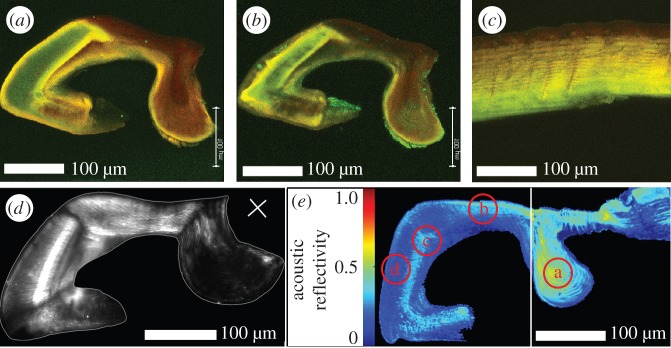
(*a*,*b*) Autofluorescence signal (maximum intensity projection) of a longitudinal section (thickness 30 µm) of the pad in (*a*) wet and (*b*) dry states. Excitation/detection emission wavelengths: 488/499–555 nm (green channel); 561/578–678 nm (red channel). (*c*) Maximum intensity projection of a longitudinal section of the metatarsal exoskeleton (thickness 30 µm) in wet state. (*d*) Polarized light microscopy image of the pad in wet state. The white line indicates the outline of the pad. The orientation of the polarizer-analyser is indicated by the white cross. (*e*) SAM image of the pad sagittal section. The picture consists of two merged images (white line) obtained from two samples measured at the same experimental conditions. The colour code indicates the reflectivity distribution for acoustic waves. Regions a–d indicate the positions chosen for nanoindentation measurements on the same samples.

CLSM was also applied to a pad section cut along the sagittal plane. The section was first measured hydrated and again after allowing it to dry for 30 min at room temperature (figure 4). The auto-fluorescence pattern of the pad section is rather complex compared to what is commonly found in the spider's exoskeleton, e.g. along the metatarsus (figure 4*c*). While the distal part of the pad is dominated by green channel fluorescence and contains an internal inner region with only weak fluorescence the dorsal part and the ‘appendix’ are predominantly showing auto-fluorescence in the red channel. Interestingly, a pronounced dark-red region in the inner part of the distal pad region is observed. Such strongly sclerotized internal cuticle is not common in arthropods, except in structures like muscle attachment sites or sclerites [1]. The level of sclerotization in the metatarsus away from the pad region decreases from exocuticle inwards towards the endocuticle following a well-known pattern common in arthropods [28,30] (figure 4*c*). After dehydration, CLSM images show large shrinkage in regions of less sclerotized cuticle (figure 4*b*), while regions of highly sclerotized cuticle retain a similar shape regardless of their water content. The largest effect is seen on the distal part of the pad, which shrinks to about half of its original size.

### 3.3. Chitin and protein distribution and alignment

**Figure 5.**
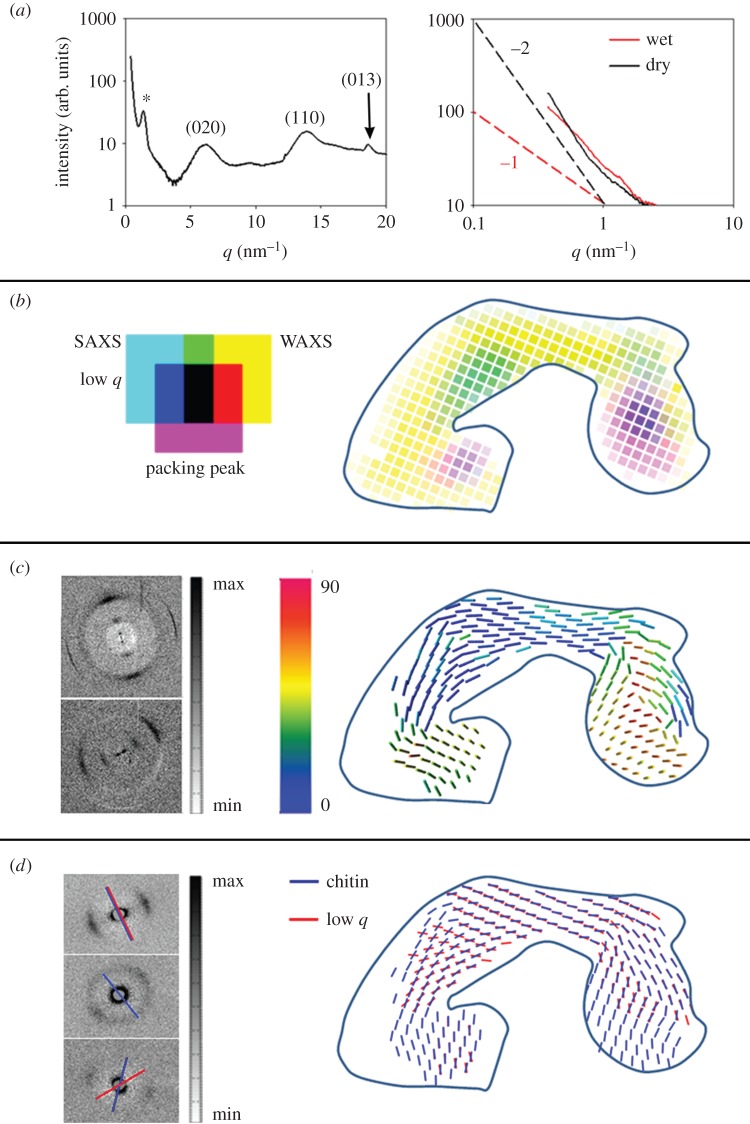
X-ray scattering analysis of the pad slice cut in sagittal plane. (*a*)(i) Characteristic pattern of radially integrated X-ray scattering measured at the dorsal part of the pad in its wet state. The pattern contains both SAXS and WAXS scattering regions. The SAXS peak around *q* = 1.3 nm^−1^ is assigned as the packing peak from chitin fibrils (marked with *). The main chitin diffraction peaks in the WAXS region are indicated. Right: SAXS region radially integrated X-ray scattering pattern plotted in a double log scale, extracted from the distal region of the pad in wet and dry states. The dashed lines represent slopes of −1 (red), and of −2 (black). (*b*) Intensity map assigning different components contributing to the X-ray scattering patterns as shown by example in (*a*). Cyan colour intensity: diffuse scattering intensity at lowest measured scattering vectors (*q* = 0.37–0.45 nm^−1^) and representing scattering from nanometre-sized cuticle components. Yellow colour intensity: integrated scattering intensity from (110) and (013) chitin diffractions (fitted peak areas), representing the distribution of chitin. Magenta intensity: the packing peak intensity (fitted peak area) in the SAXS region, representing the in-lattice ordering degree of chitin fibrils. Blue solid line indicates the outline of the pad. (*c*) Representation of three-dimensional orientation of chitin fibrils in the pad. Data analysis was based on the non-symmetric azimuthal distribution of the (110) chitin crystal peak in the WAXS region ([31]). Bars show the mean orientation of the chitin fibrils in different parts of the pad. The colour code indicates the chitin tilting angle out of the sample plane. Left: examples of the two-dimensional scattering pattern: in-plane fibres (upper) and out of plane (lower). (*d*) Orientation of the nanometre-sized objects extracted from the low-*q* signal and that of chitin fibrils extracted from the packing peak. Both orientations were determined from the non-isotropic azimuthal distribution of the respective SAXS signal. Note that only measurement points where both components show preferred orientation simultaneously are shown.

X-ray scattering measurements were performed with 30 µm thick pad slices in hydrated and dry states, using a focused beam measuring 10 µm in diameter. Structural parameters as well as an estimate of the relative content of chitin and protein in the pad material were obtained from analysing both, small- (SAXS) and wide-angle X-ray scattering (WAXS) regions (figure 5).

The signal in the WAXS region (figure 5*a*), nominally referred to the scattering region above *q* = 4 nm^−1^, is dominated by the diffraction signal from the crystalline chitin-protein fibrils (figure 5*a*). Although crystallite orientation strongly affects the WAXS intensity (see the electronic supplementary material, figure S3), integration over the scattering intensity of Bragg reflections with close to orthogonal orientation, the (110) and the (013) were used in order to gain qualitative information on the relative distribution of chitin in the pad (yellow scale in figure 5*b*). A scattering signal from chitin crystals is observed throughout the entire pad cross-section, albeit with significantly lower intensity at the distal end of the pad. In this region, the intensity of both (110) and (013) reflections is low, indicating low chitin content. In other regions with low WAXS intensity, within the ‘appendix’ and in the ventral wall of the pad, the (013) reflection is completely missing in the WAXS signal, indicating that the crystallites here are oriented mainly out of the section plane (electronic supplementary material, figure S3).

In the SAXS region, the inter-fibrillar packing peak can be used to determine the packing degree of chitin-protein fibrils and their spacing [32]. The intensity of the packing peak, related to the degree of chitin-protein fibril alignment and their orientation, is mapped in figure 5*b* (magenta scale). The packing peak intensity is largest in the ventral part of the pad, and in the central part of the ‘appendix’ (violet regions in figure 5*b*). The *q*-position of the packing peak relates to the distance *d* between the fibrils by *q* = 2*π*/*d*, where *d* is largest (4.82 ± 0.02 nm) in the centre of ‘appendix’ and smallest (4.59 ± 0.04 nm) at the outer cuticle layers. Sample dehydration results in a slight reduction of the inter-fibrillar distance, now ranging between 4.76 ± 0.03 nm and 4.52 ± 0.04 nm, respectively. Note that these values exemplify one studied specimen only. An independent measurement of another pad section in wet state revealed an inter-fibrillar distance of 4.93 ± 0.02 nm in the appendix and 4.73 ± 0.04 nm in the outer cuticle layers, respectively. Thus, although small variation between the samples exists, the distribution pattern remains the same.

### 3.4. Fibril orientation

The in-plane orientation of chitin-protein fibrils was determined from the anisotropic azimuthal distribution of the packing peak in the SAXS region (figure 5*a*). The out-of-plane angle of the fibrils on the other hand was extracted from the angular distribution of the (110) reflection of the chitin crystal in the WAXS region according to methods described in the literature [31,33,34]. The three-dimensional fibril orientation mapped in figure 5*c* shows strongly varying chitin-protein fibril orientation in different regions of the pad. The ventral side of the pad and the ‘appendix’ are dominated by out-of-plane chitin-protein fibril orientation (green-, yellow- and red-bars regions in figure 5*c*), while the dorsal and the distal parts of the pad are dominated by an in-plane organization of chitin-protein fibrils (blue-bars regions). This latter case may also arise from a plywood organization typical for spider cuticle [35]. In order to unequivocally determine the fibril orientation and microstructural arrangement, the same measurement and analysis were performed for two additional sections (assigned as transverse1 and transverse2 in figure 2*b*) cut orthogonally to the section described in figure 5 (electronic supplementary material, figures S4 and S5). For a pure plywood organization, in-plane scattering patterns are obtained regardless of the incident beam orientation within the plywood plane, whereas a parallel fibril arrangement gives an in-plane orientation signal only when viewed perpendicular to the fibril long axis. In the transverse section, in-plane fibril orientation dominates the entire sample (electronic supplementary material, figure S4*c*), indicating that the blue-bars regions in figure 5*c* are characterized by a pure plywood structure, while the green-, yellow- and red-bars regions are dominated by a parallel fibre orientation in agreement with the observations from WAXS.

Figure 5*d* maps the distribution of diffuse scattering intensity at low *q*-values (0.4 nm^−1^ < *q* < 0.6 nm^−1^). The signal at such low *q*-values arises from electron density contrast between nanometre-sized components in the sample. These may be pores, proteins or other molecules (e.g. sclerotization agents) within the spider cuticle. There are two prominent regions exhibiting high diffuse SAXS signal intensity (cyan regions in figure 5*b*). One is close to the distal end where CLSM shows a high degree of sclerotization, the other in the centre of the ‘appendix’.

The slope of the integrated scattering intensity plotted in log–log units provides more details about the geometry of the scattering objects using the Guinier analysis [36]. For the wet sample, a slope of −1 is found for measurements at the distal part of the pad and at the centre of the ‘appendix’. For the rest of the pad in the wet state, the log–log slope equals to −2. Interestingly, in the pad's dry state the scattering intensity slope in the log–log plots equals to −2 for the entire sample.

For some regions of the pad, the low-*q* SAXS signal discussed above shows pronounced anisotropy. In most of those cases, the anisotropy follows the same orientation as that of the chitin signal. However, in some regions, the orientation is mismatched with respect to chitin. The orientation of the low-*q* SAXS signal is shown in figure 5*d* together with the in-plane orientation of the chitin-protein fibrils. The region with the largest orientation mismatch between the chitin signal and the low-*q* SAXS signal is in the distal part of the pad, the same region in which we find a slope of −1 in hydrated samples.

### 3.5. Interpretation of small-angle X-ray scattering data

A SAXS slope of −2 in a log–log plot follows the Gaussian chain model of a randomly organized scattering object [37] to which we attribute the scattering of the matrix proteins [32]. If there is no interference a slope of −1 indicates cylindrical scattering objects [37]. Applying the Guinier analysis [36], we determined an average cylinder diameter of 4.7 ± 0.1 nm. The size of these objects is similar to the size of the chitin-protein fibrils determined before using the packing peak position. We therefore assume that the scattering objects here are chitin-protein fibrils. Importantly, the analysis indicates that these chitin-protein fibrils are loosely packed and not arranged in a lattice. Note, however, that in the same pad regions, we also detect the inter-fibrillar packing peak (albeit with different orientation), suggesting that two types of chitin-protein fibril arrangement occur in the same region of the pad.

From the SAXS signal anisotropy, we determine that the ‘loose’ fibrils are oriented almost perpendicular to the pad's distal surface while the tightly packed fibrils (i.e. in twisted plywood structure) follow the curvature of the pad surface. In the dorsal region and in the ‘appendix’, the orientations of both components coincide.

### 3.6. Micro-channels and lamella organization at the distal and dorsal parts of the pad

Polarized light microscopy was used to further analyse the orientation of chitin-protein fibrils in different parts of the pad. The bright regions in figure 4*d* arise from chitin-protein fibrils oriented within the plane of the sample (regions parallel to the polarization angle indicated by the white cross appear dark). In accordance with the results of X-ray scattering analysis, the dorsal and distal parts of the pad appear bright due to the contribution of the in-plane chitin-protein fibrils within the plywood structure. The ‘appendix’ region and the ventral part of the pad, however, appear dark as these regions are dominated by parallel chitin-protein fibrils oriented out of the sample plane, as concluded above from the analysis of the WAXS signal. In the distal region of the pad, we observe a striation pattern roughly perpendicular to the pad surface (in the same direction as the ‘loose’ chitin signal obtained from the analysis of the SAXS signal above).

**Figure 6.**
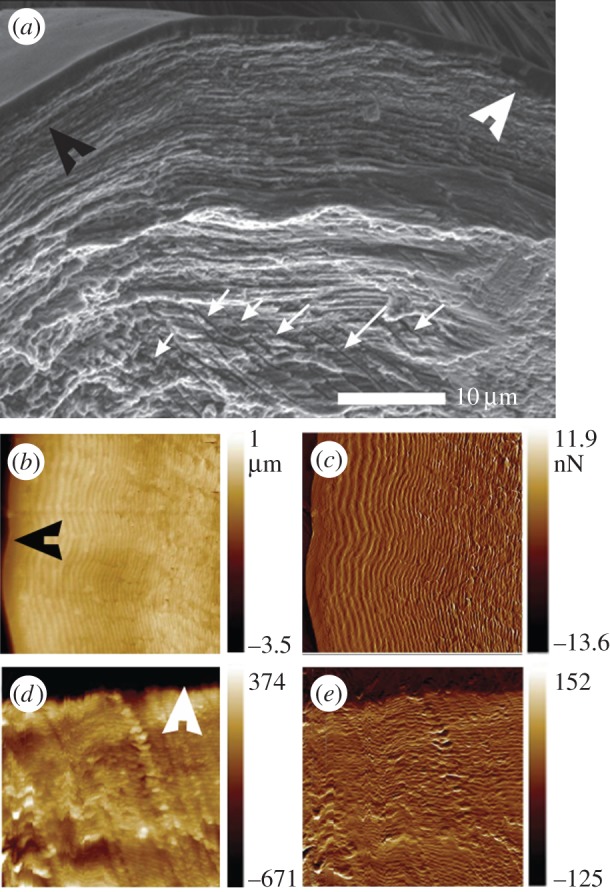
(*a*) SEM image of the dry fractured pad section (in sagittal plane). The distal and the dorsal directions are indicated by black and white arrowheads, respectively. The white arrows point to micro-channels within the chitin sub-structure of the pad. (*b*,*c*) AFM images: topography (*b*); phase (*c*) of the distal surface of the pad. (*d*,*e*) AFM images: topography (*d*); phase (*e*) of the dorsal surface of the pad.

SEM and AFM images clearly show the lamellar arrangement in the pad cuticle (figure 6). The spacing between the lamellae, which is directly related to the rotation angle of the fibrils in the plywood structure, changes gradually from the surface towards the interior of the pad. The direction of this gradient in lamella thickness changes in opposite directions distally and dorsally on the pad. Thus, on the dorsal side the spacing between two adjacent lamellae changes from approximately 250 nm at the surface to approximately 400 nm in the interior, as commonly observed in arthropod cuticles. At the distal side, the thicker lamellae are found close to the surface (approx. 680 nm), and the lamellae become thinner (approx. 300 nm) in the interior.

SEM micrographs of the distal part of the pad also show multiple channels a few micrometres in diameter, oriented perpendicular to the distal surface (figure 6*a*) which is in the same direction as the ‘loose’ chitin-protein fibrils and the striation pattern in the polarized light microscope.

### 3.7. Micromechanical characterization

SAM quantifies the reflectivity, *R*, of focused acoustic waves from the surface of the sample with a spatial resolution of about 1 µm. The measured values for *R* depend on the sample stiffness and its mass density. SAM data obtained from embedded and polished pad sections, measured under water, show the spatial distribution of *R* along the pad (figure 4*e*). The previously observed variations in sclerotization degree and chitin-protein fibril orientation correlate with the sound reflectivity; the bright-coloured regions (high reflectivity) in figure 4*e* correspond to the dark reddish (highly sclerotized) regions in figure 4*a*. In addition, the ‘appendix’ region shows a particularly high reflectivity. Since the stiffness of the fibrils is higher along their long axis, this result correlates with the occurrence of out-of-plane orientation of the fibrils in this region as determined from WAXS signal analysis. A noteworthy exception to this trend is a bright region in the centre of the pad. The bright line in the SAM image corresponds to the green-to-red transition region in the respective CLSM image. Note that in the absence of a measure of the sample mass density, we cannot extract quantitative information regarding the elastic modulus.

For quantitative determination of the elastic moduli, albeit at lower spatial resolution, we performed nanoindentation experiments under hydrated conditions on the same samples at four different regions. The regions examined are denoted by a–d in figure 4*e*. The results (table 2) show large differences of the reduced elastic modulus *E*_r_ between the regions studied. The highest value of 8.3 (±1.1) GPa was found in region a, the ‘appendix’ region, while in region b, the dorsal part of the pad, which shows a similar sclerotization level as the ‘appendix’, we measured an average value of 2.8 (±1.3) GPa. This result is consistent with SAM results and reflects the effect of fibre orientation. Note, that the elastic modulus of chitin is roughly four times higher along the *c*-direction (long axis) than along the *a/b* directions [38].

**Table 2.** Nanoindentation results from different parts of the sagittal section of the pad. The different regions are indicated in figure 4*e*.

measured region		reduced elastic modulus (GPa)
a	appendix	8.3 ± 1.1
b	dorsal part	2.8 ± 1.3
c	sclerotized internal part	1.0 ± 0.3
d	distal part	0.1 ± 0.07

The highly sclerotized internal region c of the pad, is characterized by *E*_r_ = 1.0 (±0.3) GPa. And most strikingly, the distal part, region d, shows an extremely low reduced elastic modulus of *E*_r_ = 0.1 (±0.07) GPa. Such low values are mostly known for hydrated unsclerotized cuticles of insect larvae and puparia and are uncommon in adult exocuticles [39].

## 4. Discussion of biological relevance

**Figure 7.**
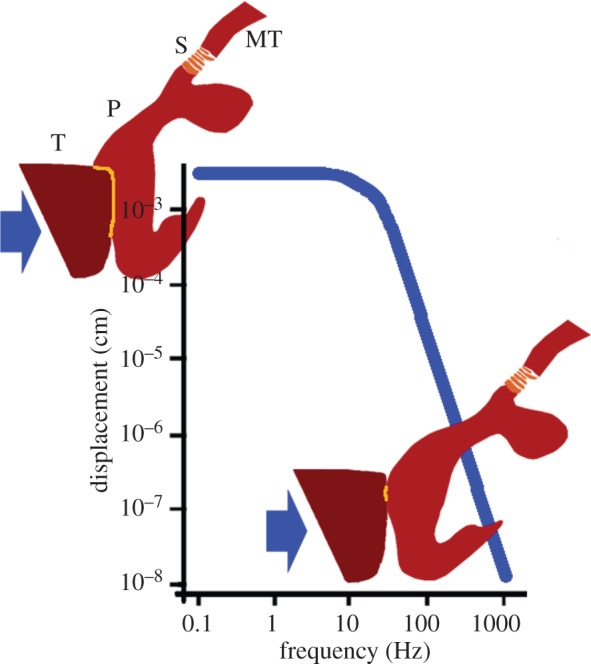
A scheme of the pad's performance in the low- and high-frequency working ranges of the metatarsal vibration sensor. Blue line: typical threshold (tuning) curve, adapted and schematized from ref. [8], showing the displacement of the substrate (and tarsus) necessary to elicit a response of the sensory cells at different frequencies. At low frequencies below *ca* 40 Hz large displacements are required. According to our present results this is because much of the tarsal (T) displacement at its contact area with the pad (P) is used to compress the pad's hydrated and soft frontal part (upper inset). At high frequencies, however, minute displacements suffice to elicit a nervous response (lower inset). Then the epicuticle on the pad's front side undergoes glass transition, implying sufficient stiffness to transmit the mechanical stimulus to the slits (S) of the vibration receptor much more effectively than at low frequencies [15]. Arrows show load direction. Yellow lines indicate the contact area between the tarsus and the pad. T, tarsus; P, pad; S, slits of the vibration receptor; MT, metatarsus.

The cuticular pad at the distal end of the metatarsus of *C. salei* plays an important role in the mechanical transmittance and transformation of stimuli which deflect the tarsus and stimulate the metatarsal vibration receptor. The sources of the organ's adequate stimulation are manifold. Stimuli primarily include the vibrations of the plant on which the spider sits and which are caused by the movements of predators or prey, as well as the vibrations actively used by the spiders to communicate during courtship. However, the metatarsal lyriform organ is also stimulated by low-frequency tarsal deflections as they occur during the spider's own locomotion and other behaviour such as prey capture and the spinning act and as they are typical of the vibrational background noise caused by abiotic sources like wind [1,40]. Upon the deflection of the tarsus, the proximal end of the tarsus pushes against the distal end of the metatarsus where the pad is located. High-frequency stimuli beyond *ca* 40 Hz are detected with high sensitivity regarding tarsal deflection amplitudes necessary to activate the sensory cells. The viscoelastic properties of the unusually thick epicuticle of the pad represent a very effective mechanical high-pass filter [15]. Low-frequency stimuli (lower than about 40 Hz) require much larger tarsal deflections in order to be detected by the vibration receptor organ. Here, we describe how the structural features of the pad allow for the transmittance of high-amplitude low-frequency stimuli and at the same time protect the sensory organ against over-compression and damage. The scheme depicted in figure 7 summarizes the pads performance at high and low frequencies.

In their natural habitat, spiders are also exposed to lateral deflection of their tarsi (i.e. off the main long axis of the tarsus–metatarsus) and their vibration receptors respond to these [1]. The crescent-like shape of the pad is well suited to allow such deflections, as can be seen in figure 3*c*. Although other shapes might also be valid (for example, a round pad), they might suffer from other drawbacks such as requiring more material at regions that are normally not activated. The elaborate morphology of the pad and, in particular, the sub-structure of the material of which it is made are discussed in more detail in the following.

### 4.1. The distal end of the pad

The distal end of the pad is extremely soft showing only 100 MPa for the reduced elastic modulus as measured in nanoindentation experiments under hydrated conditions. In agreement with this, we have also found that this pad region is highly hydrated under natural conditions and the least sclerotized part of the pad. The lamella thickness in this region decreases from the surface inwards, unlike in the rest of the leg where lamella thickness increases within the different layers (exo-, meso-, endo-cuticle) of the exoskeleton [30,35]. Lamella thickness and chitin-protein fibril orientation were recently shown to affect water sorption capability of the cuticle (C Valverde-Serrano, Y Politi, L Bertinetti 2014–2015, unpublished results) where large lamellae swell much more than thin ones per unit volume. Thus, the occurrence of thick lamellae at the distal end of the pad, as shown in figure 6*b*, also contributes to the hydration properties of this region together with the low sclerotization [28]. SAXS results show that in addition to the plywood structure that forms the lamellae seen in figure 6*b*,*c* some component occurs as loosely bound fibrils, possibly chitin-protein fibrils. Upon dehydration, the signal from these fibrils disappears, indicating that the fibrils are suspended in water or form a gel-like material (magenta regions in figure 5*b*). This signal is highly anisotropic with a directionality that follows the direction of multiple micro-channels observed using SEM (figure 6*a*) and polarized light microscopy (figure 4*d*). We therefore suggest that these channels are filled with chitin-based gel-like material, which might also account for the large swelling in this region. The micrometre-sized channels run in a direction perpendicular to the distal end surface (towards the contact region with the tarsus). Using AFM imaging, McConney *et al*. [14] reported droplets of epicuticular substance secreted from pores at the surface of the pad. We suggest that these are the openings of the micro-channels observed by us. The function of this secretion is unknown. The secretion may serve to better couple tarsus and pad during stimulus transmission, similar to insect attachment pads [41]. However, if the secretion level is high it may act in the opposite way, i.e. as a lubricant. More study is needed in order to answer this question.

The softness (100 MPa) of the pad's distal contact region with the tarsus increases the contact area with the tarsus and allows the largest deformation upon tarsal deflection while only comparatively small displacement of the pad is observed at the contact region with the slits. In this way, substrate displacements of the order of up to 200 µm, in accordance with Barth & Geethabali [8] are translated to small (non-destructive) slit compressions of only few micrometres (3.5 ± 0.7 µm at 9 ± 1° tarsal deflection), and over-stimulation, or slit damage is avoided. Thus, the pad acts as a ‘bumper’ damping the impact of large substrate displacements.

### 4.2. Internal layer of sclerotized cuticle

The interior part of the cuticle in the central region of the pad is highly sclerotized as judged from CLSM, which also endows it with a relatively high elastic modulus of 1 GPa. This specific distribution of cuticle sclerotization may imply a functional, probably mechanical role. From this region onwards proximally, the pad is no longer deformed but displaced towards the slit region. This is most likely owed to the stiffening in this region. In agreement with the present findings, Schaber *et al*. [17] found that the force needed to deflect the tarsus against the metatarsus (*ca* 1.5 mN at 4°) increases exponentially with the deflection angle. This increase of force from small deflection angles to higher ones may reflect the pad deformation behaviour related to its structure, where at small angles the distal part of the pad is easily deformed and at higher angles, the internal sclerotized region might resist further deformation. The dorsal part of the pad is also highly sclerotized and is mainly made up of twisted plywood cuticle with rather thin lamella. It thus provides bending stiffness to support pad displacement at higher loads and to restrict the displacement.

### 4.3. The ‘appendix’

The ‘appendix’ region of the pad is characterized by a particularly high level of cuticle sclerotization as well as a high degree of parallel arrangement of chitin-protein fibrils. The parallel fibrils are oriented out of the plane of the sample sagittal sections (figure 5*c*). This also explains the measured values for the reduced elastic modulus (of about 8 GPa) in this region for sagittal sections. A similar structure of parallel fibril orientation was also found in the ventral side of the pad section. From µCT analysis, we know that these two regions are connected (figure 2*g*,*h*) and could therefore form a stiff frame reinforcing the entire pad. This frame together with the unusually sclerotized internal cuticle is expected to resist further pad deformation and instead to allow small displacements of the pad towards the slits region.

Another interesting observation is that the ‘appendix’ ridge runs parallel to the slits of the lyriform organ and extends underneath them (e.g. figures 2*c*,*d*, 3*c*–*e*). We suggest that the ventral extension of the appendix helps to distribute the load to lower regions of the slits, ensuring an equal distribution of the compressive load along the long axis of the organ, perpendicular to long axis of leg and along the slit walls depth. It is tempting to speculate that this may contribute to the puzzling likeness of the nervous responses recorded from the two dendrites innervating each slit and ending at its outer and inner end, respectively [16].

## 5. Conclusion

The pad was described as a high-pass filter transferring only high frequencies with high efficiency [42]. Most of this filtering occurs at the epicuticle, the viscoelastic mechanical properties of which were recently determined using AFM at various temperatures and signal frequencies [15]. Low-frequency stimuli are damped by the epicuticle and not transferred to the slits if applied with low amplitude. However, low frequencies below about 40 Hz and even down to 5 Hz and less are detected by the sensory cells of the slits if applied with high amplitude are detected by the sensory cells of the slits [8,16]. Tarsal vertical displacements will then have to be as large as some hundreds of micrometres, corresponding to a tarsal angular tilt of approximately 10°. We propose a mechanism for the transmittance and filtering of the full frequency range of the biologically relevant stimuli via the pad to the metatarsal lyriform organ; our results suggest that during such substrate displacements, the tarsus pushes against the pad and deforms it by some tens of micrometres at its most distal, highly hydrated region. Beyond this region, the sclerotized region and the supporting frame (appendix and ventral pad region) resist the deformation and are displaced to push against the slits. Now, however, the displacement values are considerably scaled down to only few micrometres. In addition, the ‘appendix’ running parallel and along the slits ensures uniform compression roughly perpendicular to the long axes of the slits for stimuli deflecting the tarsus vertically or laterally.

Taken together with previous results on high-pass filtering properties of the pad, our results emphasize the functionality of the pad transmitting selected low-frequency stimuli and thus expanding its stimulus-frequency working range. The ability of protecting a sensor from being overloaded and at the same time keeping it very sensitive (slit compression in the 1 nm range) in the relevant ranges of frequency and intensity, as well as, proper tuning of signal-to-noise ratios, while being embedded in a stiff material, is well demonstrated by the pads structure and design.

Unravelling the structural arrangement in such specialized biological structures may provide new construction principles for the design of new types of embedded artificial microsensors and microactuators for a broad range of applications [43]. Such bioinspired embedded sensors might be of interest for micro-electro-mechanical systems (MEMS) and Bio-MEMS technologies, for micro- and nanorobotics, vibration control and sensing fabrics [23,44,45]. Other examples of bioinspired strain sensors for application in robotics and for various microdevices take inspiration from insect campaniform sensilla [46,47], which are analogous structures to the slit sensors in spiders. Recently, crack-based ultrahigh sensitivity strain and vibration sensors inspired by the spider slit sense organs, have been designed and constructed [48]. Comprehensive understanding of biological sensing systems can help not only developing artificial sensors, but will also shed light on the biological and ecological adaptive advantage of such sensing mechanisms.

## Supplementary Material

Supplementary material
