# Supplementary material for: Micro- and nano-structural details of a spider's filter for substrate vibrations: relevance for low-frequency signal transmission
Source: J R Soc Interface. 2015 Mar 6;12(104):20141111. doi: 10.1098/rsif.2014.1111 (PMC4345480; doi:10.1098/rsif.2014.1111)
Supplement: Supplementary material [file rsif20141111supp1.pdf]

Supplementary material

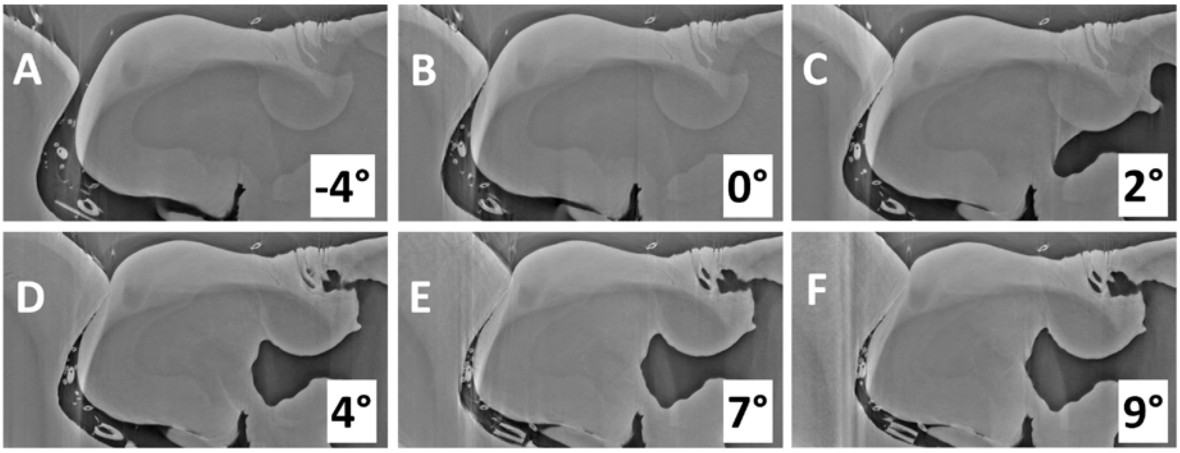

SM Figure 1: µCT virtual slices along the long-axis of the leg (sagittal plane). The slices A-F show the same slice upon different deflection angles of the tarsus and metatarsus. The angle values are related to the angle of the first contact (=0°).

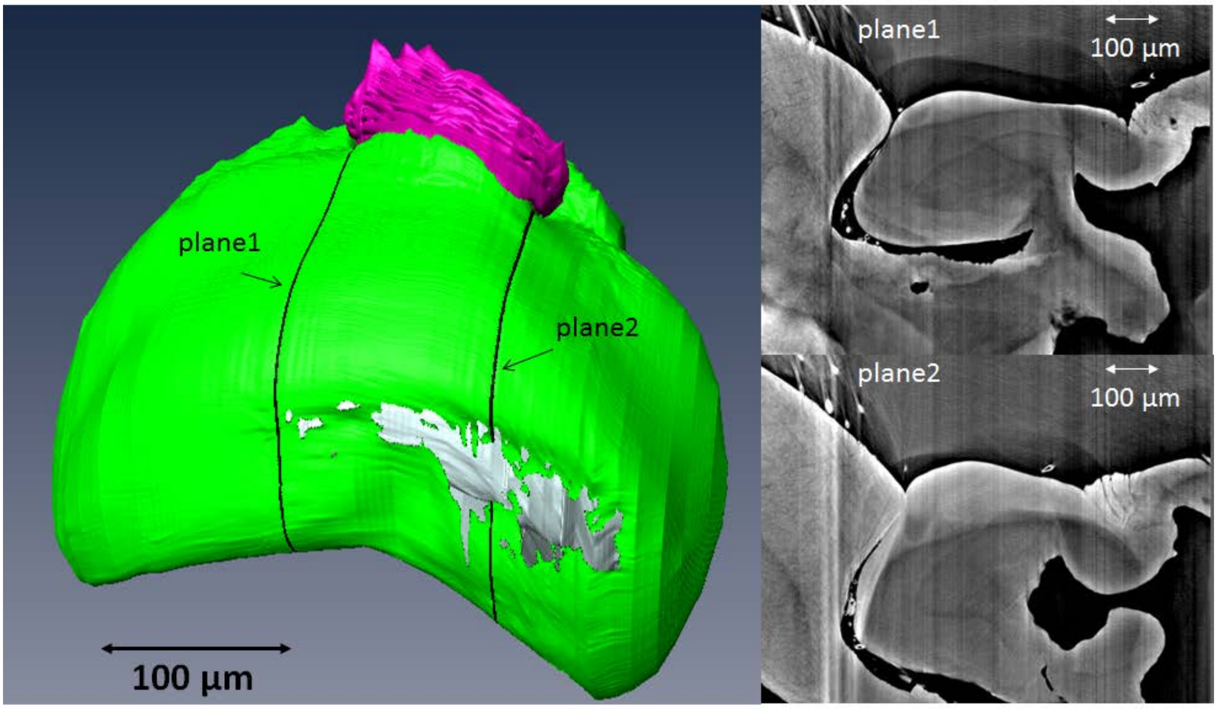

SM Figure 2: Three-dimensional shape of the pad under upon tarsal loading including a slight lateral component as shown in Figure 3. The non-symmetric deformation of the pad is demonstrated by two virtual slices at planes indicated by the black lines and shown on the right. The greyscale contrast of the images was adjusted improving the visualization of the pad contact region with the tarsus.

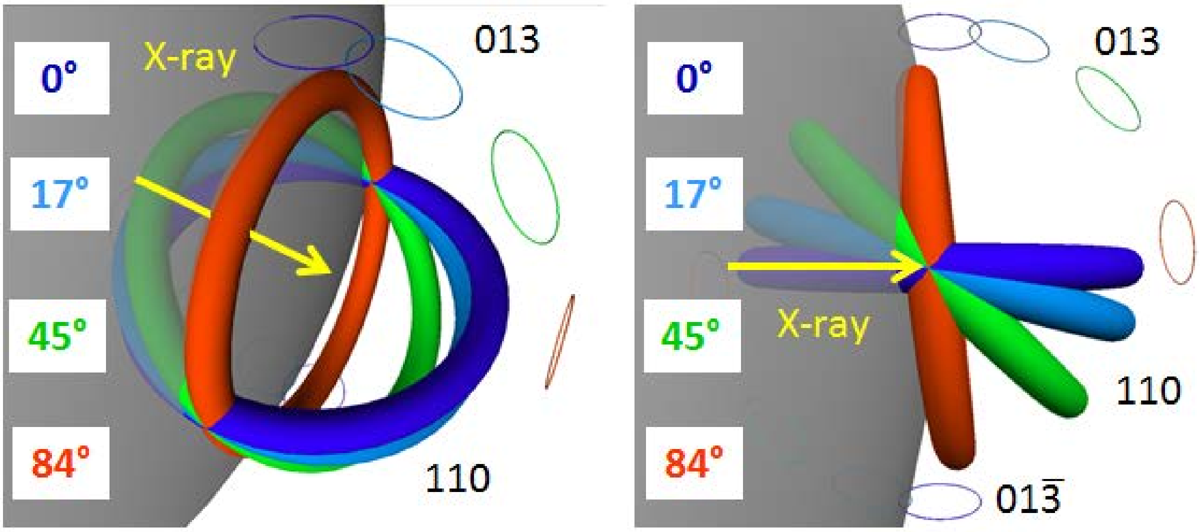

SM Figure 3: Perfect alignment of all chitin crystallographic axes yields three sharp Bragg spots in the reciprocal space, which correspond to the 110, 013 and $01\bar{3}$ reflections, respectively. Random orientation of the fibrils around their longitudinal c-axis leads to cylindrical smearing of the three spots to thee rings. The reflections are broadened due to the small crystallite size. Here the schematic view of the reciprocal space is shown including the Ewald sphere (grey) and four arbitrary chosen cases of chitin fibril orientation with respect to the X-ray beam. For each of the four chosen orientation angles (same color code as in Figure 5B) the three rings represent three Bragg reflections from chitin fibrils, i.e. 110, 013 and $01\bar{3}$, respectively. If the rings do not intersect the Ewald sphere simultaneously they do not appear in a single diffraction pattern. For gradual rotation of the fibrils in the plane of the X-ray beam (plywood structure where the normal vector is pointing perpendicular to the X-ray beam) the 110 rings are smeared to a sphere, and those for 013 and $01\bar{3}$ to a ring. The corresponding diffraction pattern appears qualitatively the same as for in-plane fibrils (tilt angle of 0°).

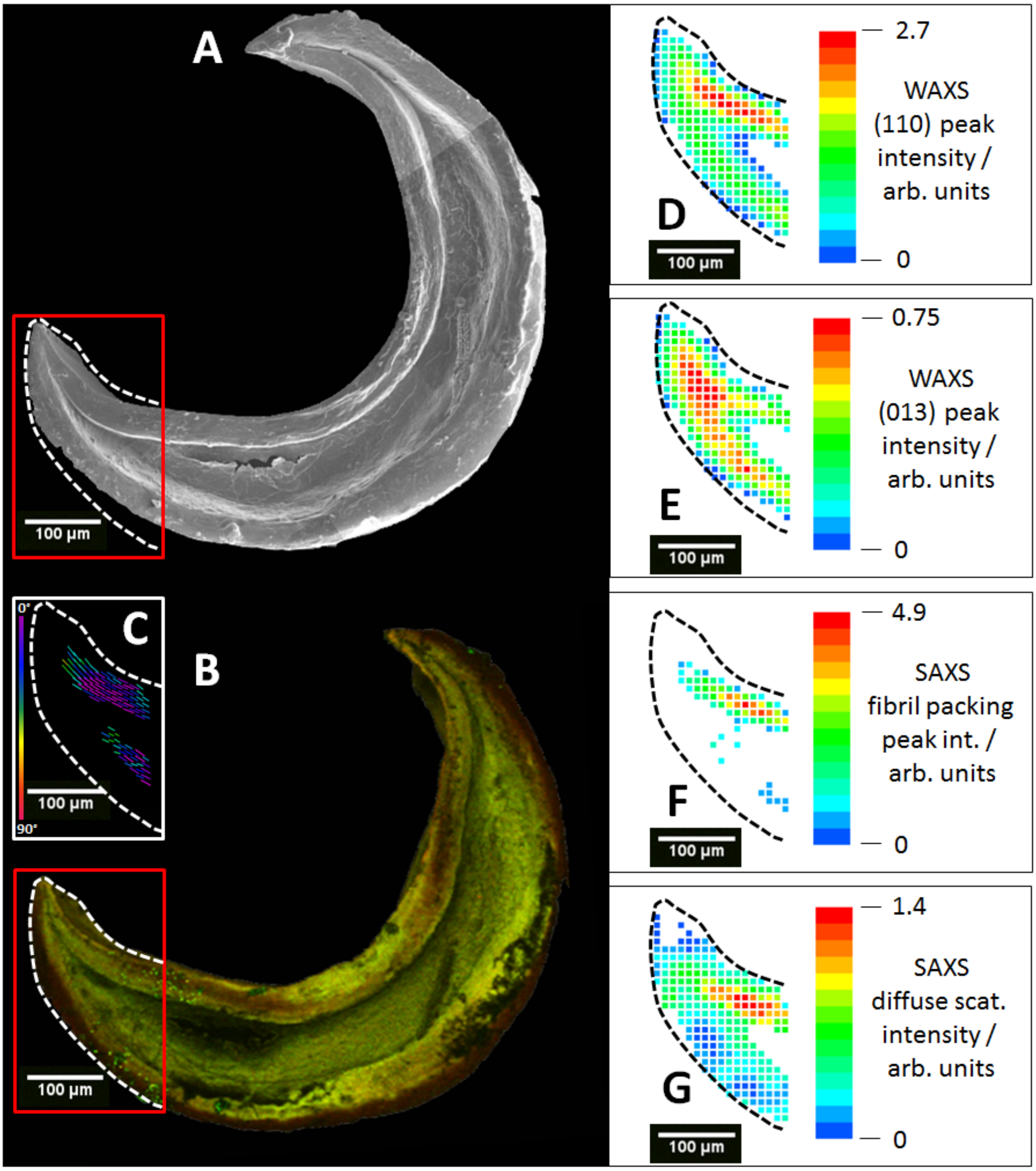

SM Figure 4: (A) SEM image of the dry transverse section slice (30 µm thickness) extracted from the middle part of pad as indicated in Figure 2 (transverse2). (B) Autofluorescence signal (maximum intensity projection) of the same sample in its wet state. Excitation / detected emission wavelengths: 488 nm / 499-555 nm (green); 561 nm / 578-678 nm (red). The two signals are superimposed. Lens / numerical aperture / immersion / image format (pixel): 20x / 0.7 / air / 1024 x 1024. (C) Three-dimensional orientation of the chitin fibrils in the sample represented by colored bars, color code indicates the tilting angle out of the plane. (D-E) Intensity distribution of the (110) chitin crystal peak (D), (013) chitin crystal peak (E) fibrils packing peak (F), and diffuse SAXS (G) in the X-ray scattering data measured for the same sample in wet conditions.

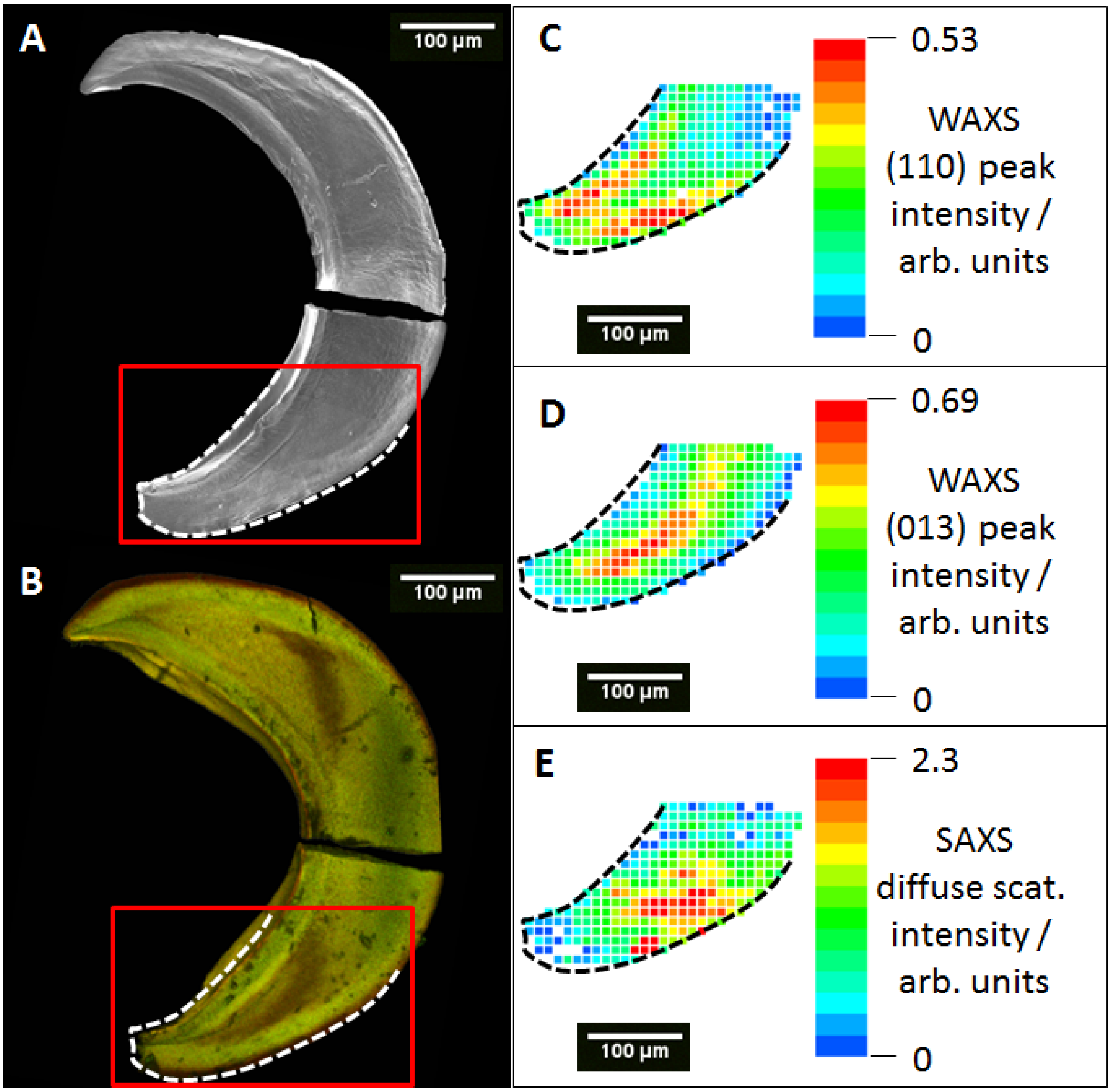

SM Figure 5: Unfortunately, the sample broke in two pieces after remounting from the sample holder for X-ray scattering experiments. In order to give an appropriate visual illustration of the sample, the two pieces were measured in SEM and CLSM under same conditions, and the respective images were merged together.

(A) SEM image of the dry long-section slice (free-standing section with 30 µm thickness) extracted from the front part of pad as indicated in Figure 2 (transverse1).

(B) Autofluorescence signal (maximum intensity projection) of the same sample in wet state. Excitation / detected emission wavelengths: 488 nm / 499-555 nm (green); 561 nm / 578-678 nm (red). The two signals are superimposed. Lens / numerical aperture / immersion / image format (pixel): 40x / 0.75 / air / 1024 x 1024.

(C-E) Intensity distribution of the (110) chitin crystal peak (C), (013) chitin crystal peak (D), and diffuse SAXS scattering (E) in the X-ray scattering data measured for the same sample in wet conditions.
